# Development of *Necrobia ruficollis* (Fabricius) (Coleoptera: Cleridae) under Different Constant Temperatures

**DOI:** 10.3390/insects13040319

**Published:** 2022-03-24

**Authors:** Yinghui Wang, Liangliang Li, Gengwang Hu, Chengtao Kang, Yi Guo, Yanan Zhang, Yu Wang, Jiangfeng Wang

**Affiliations:** Department of Forensic Medicine, Soochow University, Ganjiang East Road, Suzhou 215000, China; 20194221064@stu.suda.edu.cn (Y.W.); 20214021012@stu.suda.edu.cn (L.L.); 20214221010@stu.suda.edu.cn (G.H.); 20214221013@stu.suda.edu.cn (C.K.); 20214001021@stu.suda.edu.cn (Y.G.); 20204221029@stu.suda.edu.cn (Y.Z.)

**Keywords:** forensic entomology, in vivo measurement, *Necrobia ruficollis*, development, instar determination

## Abstract

**Simple Summary:**

Sarcosaprophagous beetles are significant pests, but they are also key indicator insects for postmortem interval estimation. In this paper, the development of *Necrobia ruficollis* (Fabricius, 1775) at five constant temperatures between 22 °C and 34 °C was investigated. The developmental threshold temperature and thermal summation constant were estimated, the relationship between the larval body length with development time, and the widths of the head capsules and the distance between the urogomphi at different instars were studied. The results provide important basic developmental data for using *N. ruficollis* to estimate the minimum postmortem interval.

**Abstract:**

*Necrobia ruficollis* (Fabricius, 1775) (Coleoptera: Cleridae) is an important cosmopolitan storage pest, and also frequently appears on highly decomposed and skeletonized corpses. It is a forensically important species expected to indicate a longer postmortem interval (PMI). Therefore, we investigated the development of *N. ruficollis* at five constant temperatures between 22 °C and 34 °C. Under temperatures of 22, 25, 28, 31, and 34 °C, the mean (±SD) developmental durations from eggs to adults were 93.00 ± 1.63, 70.67 ± 0.94, 65.33 ± 3.40, 47.33 ± 0.94, and 56.66 ± 8.73 days, respectively. According to the developmental time and accumulated degree hours results, an isomorphen diagram and thermal summation model were generated. The calculated values of developmental threshold temperature and accumulated temperature constant were estimated by a linear model to be 14.51 ± 0.52 °C and 684.12 ± 33.85 degree days, respectively. Lower developmental thresholds, intrinsic optimum temperature, and upper lethal developmental threshold temperature were estimated by a nonlinear model to be 14.61, 25.90, and 34.94 °C. Morphological indexes of larvae were obtained by in vivo measurements. A growth curve and an equation of the relationship between development time and body length were simulated. In addition, the widths of the head capsules and the distance between the urogomphi of larvae at different instars were determined by cluster analysis. Classifiers were created and validated by linear discriminant analysis. These results provide important basic developmental data for using *N. ruficollis* to estimate the minimum postmortem interval (minimum PMI). However, this study was only conducted under constant temperature, and the applicability of these data to variable temperature conditions needs to be further confirmed.

## 1. Introduction

Forensic entomology provides the scientific basis for postmortem interval (PMI) estimations through the development and succession patterns of sarcosaprophagous insects [1], and its feasibility has been verified by a large number of practical cases [2,3]. At present, the method based on insects is one of the most accurate for estimating the PMI of a corpse in the first 1–2 months of death [4]. This is because the application of forensic entomology at present is mainly based on fly studies [5,6,7], since the time required for flies to develop one generation is typically within 1–2 months [8,9,10,11].

Sarcosaprophagous beetles are one of the other important groups of insects that share corpse resources with flies [12,13], and they are also key indicator insects for PMI estimation [5]. Sarcosaprophagous beetles colonize the corpse at a relatively late stage [5,14]. Some species belonging to the families Silphidae and Staphylinidae [12,15], arrive at a corpse on the 2nd–3rd day (i.e., at an early stage of decomposition). Most species in the families Dermestidae, Cleridae, Nitidulidae, and Scarabaeidae, do not appear on the corpse until a late stage of decomposition or even at the skeletonization stage [14,15,16,17]. In addition, under the same conditions, the development time of sarcosaprophagous beetles is usually much longer than that of flies [18]. When all the flies at one scene of death have accomplished one generation of development (with only empty puparia remaining), the sarcosaprophagous beetles on the corpse can still be at the pupal stage, and can therefore be used to estimate PMI [4,19]. This suggests that research on sarcosaprophagous beetles could not only expand the PMI time window, which is currently determined by flies, but also improve the accuracy of PMI estimation in the “post-fly stage”, provide a scientific basis for PMI estimations of highly decomposed and skeletonized corpses, and finally, used together with the fly indicators to provide more accurate PMI results for corpses at the early and middle stages of decomposition [18].

Previous studies on sarcosaprophagous beetles have mainly focused on insect succession [5,14,16,20,21], but there are relatively few studies on their development in relation to PMI estimation. However, in recent years, the number of studies on the growth and development of sarcosaprophagous beetles has increased, and dozens of species in the families Silphidae [22], Staphylinidae [23,24,25], Nitidulidae [26], Dermestidae [27,28], Leiodidae [29], and Histeridae [30] have been examined. They provided important basic developmental data, including developmental duration, developmental accumulated temperature, larval body length changes, and instar discriminant parameters for related species used for PMI estimation, while isomorphen diagram, isomegalen diagram, and thermal summation models were established for the convenience of application. Simultaneously, research has also investigated the effects of drugs [31], foods [32,33], in vivo measurement method [34], and pupation substrate [33,35] on the growth and development of different sarcosaprophagous beetles, as well as the developmental rate differences between different sexes [25]. Although a lot of research has been conducted, basic developmental data are still missing on a large number of important sarcosaprophagous beetles. 

*Necrobia ruficollis* (Fabricius, 1775) (Coleoptera: Cleridae) is mainly characterized by a metallic blue to blue-green color, except for the pronotum, wings base, sternum, and legs which appear reddish brown. It mainly damages dried meat, animal medicinal materials, fur, dried fish and fish meal, cocoons, as well as cotton clothes [36,37,38], and is a globally distributed storage pest. Despite its destructive nature, *N. ruficollis* is also an important species in forensic science [38] and has been recorded in death cases and insect succession studies in many areas around the world [15,16,20,39,40]. Previous succession studies have indicated that *N. ruficollis* mainly appear on carcasses during the advanced decay stage and skeleton stage [5,16,40]. However, when the temperature is relatively higher, the adult beetles also appear on carcasses at the bloating stage [15]. In some areas, *N. ruficollis* frequently appears together with *Necrobia rufipes* (De Greer, 1775) (Coleoptera: Cleridae) [5]. After arriving at a corpse, the adult *N. ruficollis* mainly feeds on corpse tissues, but also preys on other insects on the corpse [38]. The larvae mainly feed on the decomposed corpse. Mature larvae usually pupate in the pupal chamber [41]. Since *N. ruficollis* is an important storage pest of significant forensic value, research on the developmental pattern of *N. ruficollis* has significance for both pest control and estimation of the minimum PMI. 

At present, research on *N. ruficollis* mainly includes mitochondrial whole genome analyses [38], cytological detection [42], descriptions of biological and morphological properties [41], as well as damage and control in relation to stored products [36,37]. However, basic developmental data for pest control and forensic entomological applications are still insufficient. The results of the existing succession studies showed that *N. ruficollis* mainly appears on the corpse at the late stage of decomposition or even the skeletonization stage, and is one of the insects that arrive at the corpse last [5,16,40]. Study on *N. rufipes*, a closely related species of *N. ruficollis*, has suggested that certain morphological indexes, including head capsule width, pronotum width, and mesonotum width could be used for the determination of larval instars [43]. Thus, we supposed that such indexes may also be used to determine larval instars of *N. ruficollis*. In view of this, we conducted a study at five constant temperatures between 22–34 °C to determine (1) the developmental duration and thermal summation constant of the *N. ruficollis*; (2) whether the variation of larval body length can be used to estimate the PMI; and (3) whether two indexes, head capsule width and distance between urogomphi, can feasibly be used to estimate the larval instar, in order to better use this species to estimate the PMI.

## 2. Materials and Methods 

### 2.1. Colony Establishment

Individuals of 20 *N. ruficollis* adults were collected from a pig carcass in field succession experiments in Suzhou (31°21′ N, 120°53′ E) during the summer of 2020. The adults were placed under a Zeiss 2000-C stereomicroscope (Carl Zeiss, Jena, Germany) for identification using the adult identification keys of Zhang [44], and they were placed into a 24 cm × 16 cm × 10 cm insect rearing box for feeding, which had a nylon net on the top of the box for ventilation. The insect rearing box was placed in a microenvironment incubator (LHP-300H, Yingmin Co., Ltd., Suzhou, China) at a temperature of 25 °C, a humidity of 70%, and a photoperiod of L12:D12. The feed for the adults was dried pork slices, and a water source was provided by spraying water onto sponge blocks (10 cm × 6 cm × 2 cm). Three-layer sterile non-woven masks were cut into long strips (9.5 cm × 3 cm), moistened with water, and placed into the rearing box as the substrate for oviposition. After eggs were laid, the non-woven fabric was taken out, together with the eggs and placed into a new rearing box. Observations were conducted daily. After hatching, smashed lean pork slices and soaked sponge blocks were put in. In the pre-experiments, it was found that when sandy soil, cotton, wood, and paper tissue were provided as the pupation substrate for the post-feeding larvae, they preferred to pupate in cotton. Therefore, cotton was used as the pupation substrate in this study. After eclosion, the adults continued to be fed and the adult colony was kept to at least 50 individuals for the following experiments. 

### 2.2. Observations of Development Duration

Fifty adults were placed into five insect rearing boxes at a female/male ration of 1:1, and the number of adults in each box was ten. Each feeding box was placed into an incubator set to either 22, 25, 28, 31, or 34 °C, with a humidity of 70%, and a photoperiod of L12:D12. Dried pork slices were used as adult food and soaked sponge blocks were used to provide water. Three-layer non-woven strips were wetted with water to induce adults to lay eggs. The eggs laid within 24 h were taken out of the feeding boxes and were put into plastic Petri dishes with a diameter of 12 cm, placed into incubators set to the same temperatures as before, and observed every 4 h for egg hatching. After hatching, 20 larvae were carefully transferred into 20 Petri dishes with a diameter of 6 cm to allow each larva to be raised separately. The larvae were fed with smashed dried lean pork slices and a soaked sponge block was used to provide water. The growth and ecdysis of larvae were observed and recorded every 24 h under a stereomicroscope. In this study, the larval exuviate was observed to determine whether the larvae had entered the next developmental stage. After the larvae entered the post-feeding stage, a cotton ball (8 mm × 4 mm × 1 mm) was placed into each Petri dish as the substrate material for the larvae. When the larvae formed pupal chambers in the cotton balls, the pupal chamber was observed every 24 h by illumination with the transmission light of stereomicroscope; the shadow cast by the insects was used to determine whether the larvae had pupated. There were two bases for judgement: (1) the larval body length would be significantly shortened after they became pupae; and (2) the pupa would quickly twist its abdomen, while post-feeding larvae close to pupation were almost inactive.

After pupation, tweezers were used to carefully cut a small opening in the pupal chambers and the eclosion status of adults was observed and recorded every 24 h. The minimum times taken for hatching, first ecdysis, second ecdysis, third ecdysis, pupal chamber formation, pupation, eclosion, and the time taken for 50% of the individuals to complete development, as well as the time for all individuals to complete specific development stages, were recorded. The experiment at each temperature was repeated three times using different incubators. 

### 2.3. The Determination of Larval Morphological Indexes

The above method was used to induce adults to lay eggs, and the collected eggs were raised in the incubators at 22, 25, 28, 31, and 34 °C with the humidity maintained at 70%, and a photoperiod of L12:D12 h. After hatching, the 20 larvae were raised separately in single Petri dishes and supplied with smashed dried pork slices and water. Five larvae were randomly selected each day and placed under the stereomicroscope and a Nikon D700 camera (Nikon Crop., Tokyo, Japan) was used to take photographs and collect the morphological indexes. Before pressing the shutter button, the larva had to be in the center of the camera’s field of view. The larvae exhibited body extension and contraction when crawling. Therefore, multiple photographs were taken of each larva until a high-resolution photograph showing full extension of the body length was obtained. The same method was used for obtaining the photographs of all morphological indexes to reduce the influence of parallax on these results. Sampling and photographing were stopped when the larvae began to drill into the cocoon chamber of the cotton. The experiment at each temperature was repeated three times in different incubators. 

**Figure 1:**
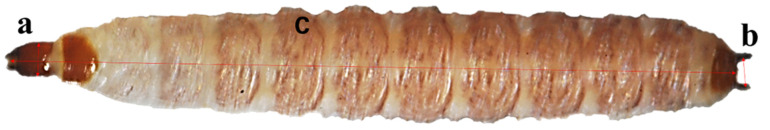
A schematic diagram of *Necrobia ruficollis* larval measurement. The two-way arrow highlights the indicators measured in this study, wherein (**a**) represents head capsule width, (**b**) represents distance between urogomphi, and (**c**) represents larval body length.

ImageJ software was used to measure three groups of data including larval body length (c), head capsule width (a), and cercus spacing (b), as shown in Figure 1.

### 2.4. Data Analysis

R3.5.2 (https://www.r-project.org/ (accessed on 19 March 2002)) and Origin Pro 8.6 (https://www.originlab.com/ (accessed on 19 March 2002)) were used for data analysis. The revised linear regression model proposed by Ikemoto and Takai [45] was used to establish the thermal summation model at different development stages. The slope and intercept of the equation in this established model were developmental threshold temperature (D_0_) and accumulated temperature constant (K) at each development stage, respectively. 

We also estimated the parameters for different developmental stages of *N. ruficollis* according to the curvilinear thermodynamic model (Optim SSI) of Shi et al. [46]. The SSI model expression is as follows:

$$r\left(T\right)=\frac{\rho_{\emptyset }\;\frac{T}{T_{\emptyset }}\exp\left[\frac{\Delta H_{A}}{R}\;\left(\frac{1}{T_{\emptyset }}-\frac{1}{T}\right)\right]}{1+exp\left[\frac{\Delta H_{L}}{R}\;\left(\frac{1}{T_{L}}-\frac{1}{T}\right)\right]+exp\left[\frac{\Delta H_{H}}{R}\;\left(\frac{1}{T_{H}}-\frac{1}{T}\right)\right]}$$

where *r*: mean development rate (1/day); T: absolute temperature (K) (273.15 K = 0 °C); *R*: gas constant (1.987 cal/deg/mol); Δ*H_A_*: enthalpy of activation of the reaction that is catalyzed by the enzyme (cal/mol); Δ*H_L_*: change in enthalpy associated with low temperature inactivation of the enzyme (cal/mol); Δ*H_H_*: change in enthalpy associated with high temperature inactivation of the enzyme (cal/mol); *T_L_*: temperature at which the enzyme is active at 50% because of low temperature (K); *T_H_*: temperature at which the enzyme is 50% active because of high temperature (K); *T_Φ_*: intrinsic optimum temperature at which the probability of the enzyme being in the active state is maximal (K); and *ρ_Φ_*: development rate at the intrinsic optimum temperature *T_Φ_* (1/day) assuming no enzyme inactivation. The value of each parameter was estimated by R 3.5.2 using the program developed by Shi et al. [46].

Regression analysis was applied to evaluate the relationship between the time after hatching and larval body length, and equations for estimating the larval age through body length were established.

In this research, the head capsule width (shown in Figure 1a) and distance between urogomphi (shown in Figure 1b) were used as cluster analysis elements for larval instar estimation. The classifier was obtained and verified by linear discriminant analysis, and the following equation was used for calculating classification value: *y* = *x*1 × *f*1 + *x*2 × *f*2. In this equation, *y* represents the classification value of age, *x* represents the weight of each measured value, and *f* represents the measured value. In addition, the statistical description values of head capsule width and distance between urogomphi at each instar were calculated. 

## 3. Results

### 3.1. Developmental Duration of N. ruficollis and Isomorphen Diagram

In our study, *N. ruficollis* was able to mate, lay eggs, and complete the whole development process at the temperature range of 22–34 °C set in this experiment. At the temperature range of 22–31 °C, with an increasing temperature, the total developmental duration of *N. ruficollis* was shortened from 92.00 ± 2.94–96.33 ± 0.94 days at 22 °C to 42.33 ± 2.05–57.00 ± 1.63 days at 31 °C. When the temperature was increased to 34 °C, the developmental duration of *N. ruficollis* was extended in comparison to that at 31 °C and was observed to be 52.33 ± 7.93–59.00 ± 7.12 days (Table 1).

**Figure 2:**
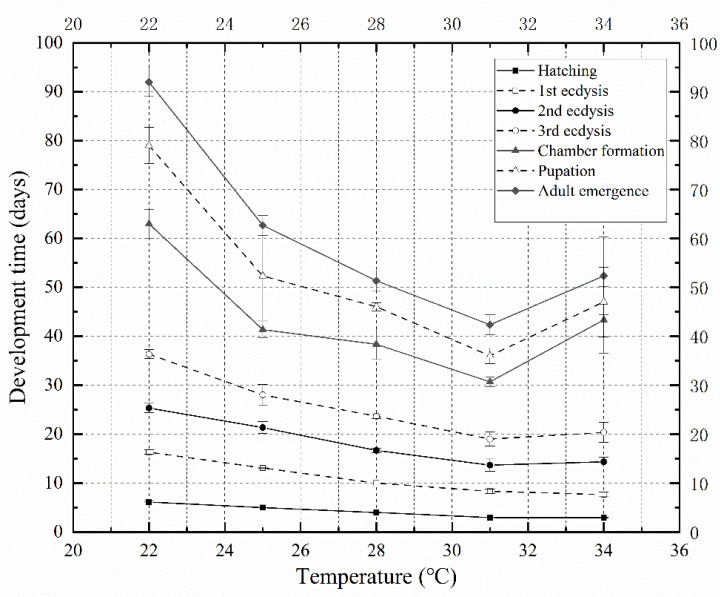
Isomorphen diagram of *Necrobia ruficollis*. The duration of each development event (hatching, 1st ecdysis, 2nd ecdysis, 3rd ecdysis, chamber formation, pupation, and adult emergence) is plotted against the time from oviposition to the onset of each event. Each curve corresponds to a particular development event. The error bar represents the SD.

According to the minimum developmental time data at each stage in Table 1, an isomorphen diagram (Figure 2) showing the development time (y-axis) required for different developmental events at each temperature (x-axis) was generated. In the temperature range of 22–31 °C, with an increasing temperature, the time required for each developmental event was gradually shortened, and the spacing between each curve also gradually shortened. When the temperature was set at 34 °C, the time required for 2nd ecdysis, 3rd ecdysis, chamber formation, pupation, and adult emergence was prolonged, and the curves showed a different trend to that in the temperature range of 22–31 °C.

### 3.2. Thermal Summation Constant and Developmental Threshold Temperature of N. ruficollis

**Figure 3:**
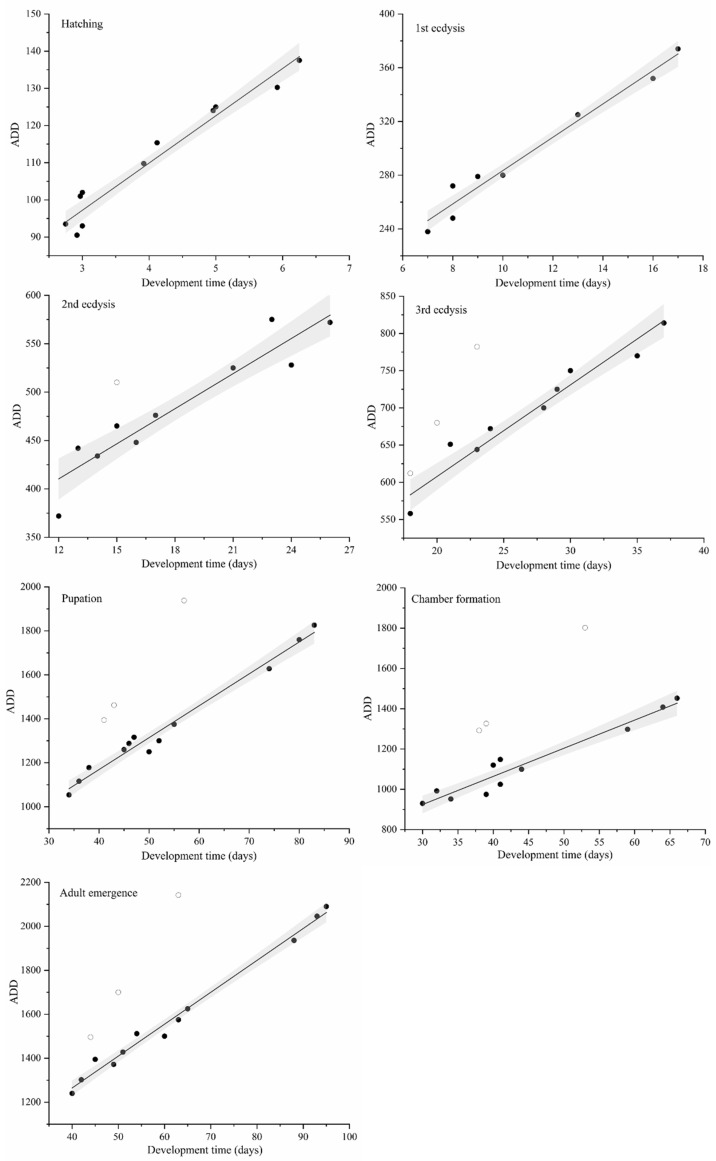
Thermal summation models of different development stages of *Necrobia ruficollis.* ● represents the data used in the regression analysis, while ○ represents the eliminated data. The solid line represents the regression line. The gray area represents the 95% confidence interval.

According to the linear regression analysis of the relationship between the time taken (x-axis) and accumulate temperatures (accumulated degree days, ADD) (y-axis) for the seven development stages, thermal summation models were established (Figure 3) and the developmental threshold temperature and thermal summation constant of each development stage were obtained (Table 2). During complete development, the development threshold temperature (D_0_) of *N. ruficollis* was 14.51 ± 0.52 °C, and the thermal summation constant (K) was 684.12 ± 33.85 degree days. Except for the determination coefficient value (R^2^) of the accumulated temperature model at second ecdysis (0.90), the R^2^ values of the thermal summation models all exceeded 0.94, which indicated that the fitting of the linear model was good. At 34 °C, the ADD data and the development time of 2nd ecdysis, 3rd ecdysis, chamber formation, pupation, and adult emergence had poor linear correlation with other temperatures and therefore, in accordance with the suggestions of Ikemoto and Takai [45], these data were eliminated in the regression analysis.

**Figure 4:**
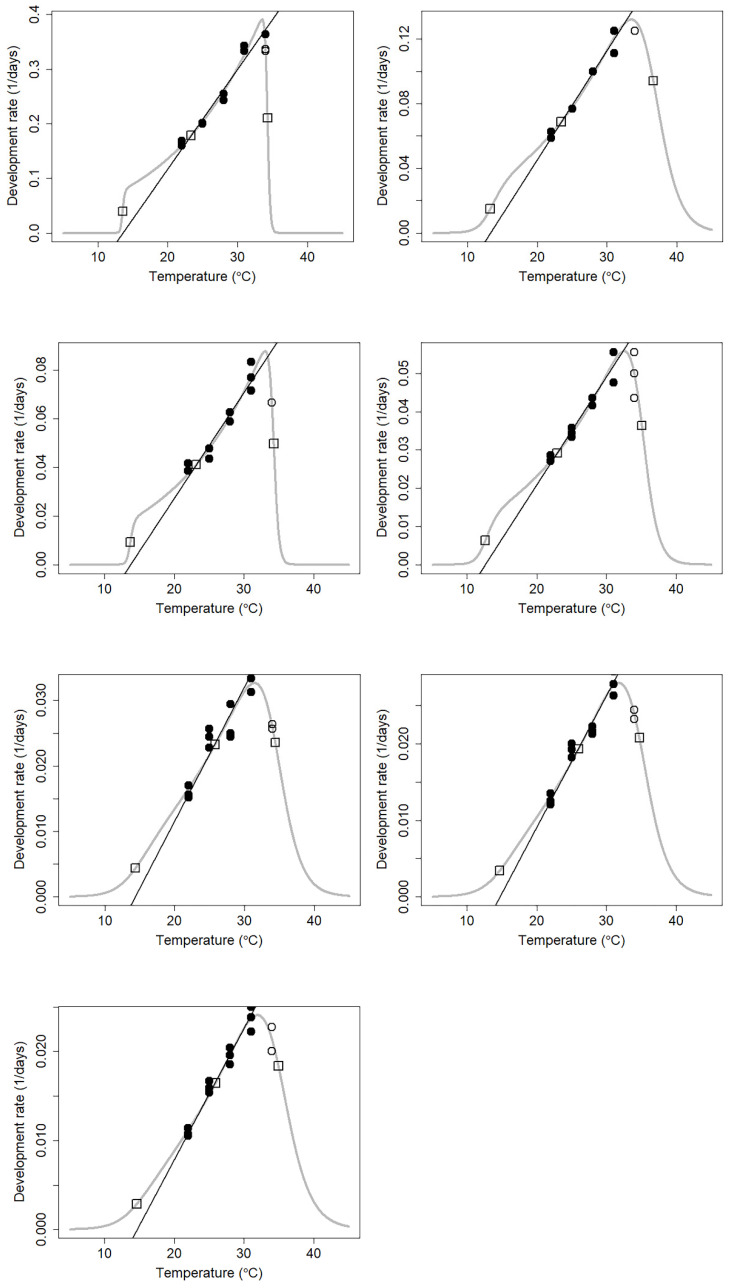
Nonlinear thermodynamic Optim SSI models of the seven developmental stages of *Necrobia ruficollis* circles indicate data points. The curved line indicates the developmental rate predicted by the Optim SSI model of Shi et al. [46]. The three open squares denote the predicted mean developmental rates at *T_L_*, *T_Φ_*, and *T_H_*. The black circles denote the data used for the linear fitting by the reduced major axis, whereas the white circles were the data excluded from the linear fitting.

Subsequently, we used the thermodynamic nonlinear Optim SSI model to fit the data at 22–34 °C (Figure 4). The lower developmental threshold, intrinsic optimum temperature, and upper lethal developmental threshold temperature estimated by the nonlinear model were 14.61, 25.90, and 34.94 °C, respectively (Table 3).

### 3.3. The Larval Body Length Changes in N. ruficollis and the Isomegalen Diagram

**Figure 5:**
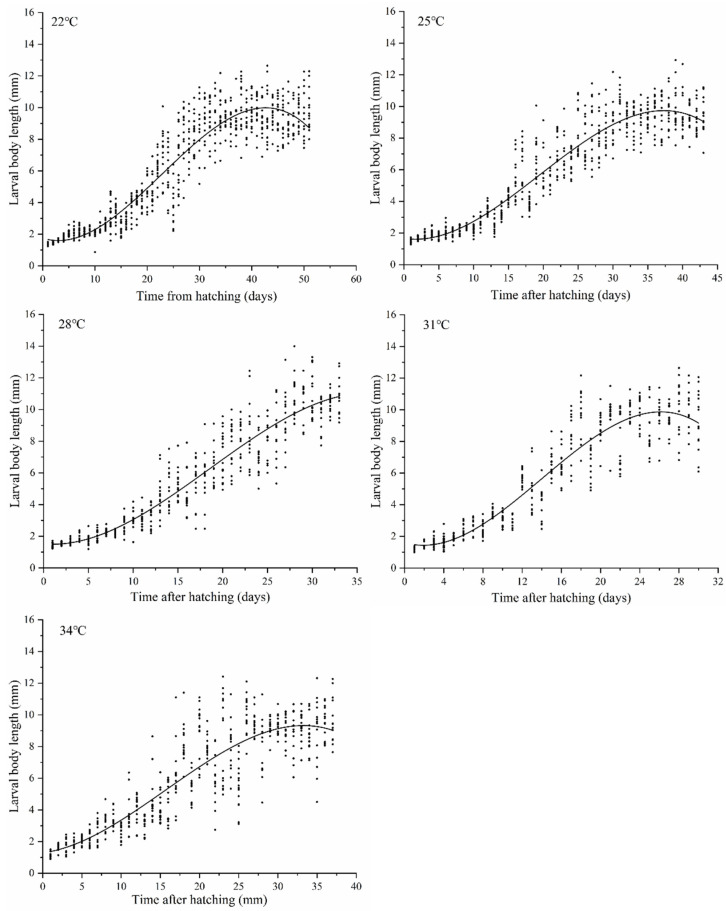
Larval body length of *Necrobia ruficollis* changes with time at five constant temperatures.

The larval body length changes from hatching to pupal chamber formation at different temperatures are shown in Figure 5. A regression analysis was conducted with larval body length as the independent variable and time after hatching as the dependent variable, and simulation equations for larval body length changes with time at five constant temperatures between 22 and 34 °C were generated (Table 4). The growth curve of *N. ruficollis* larvae represented an S-shape, including two phases of growth (namely the feeding stage) and a contraction phase (namely the post-feeding stage). At constant temperatures of between 22 and 31 °C, the development rate of larvae accelerated with the increasing temperature. However, when the temperature was at 34 °C, the larval development rate slowed down compared to that at 31 °C.

**Figure 6:**
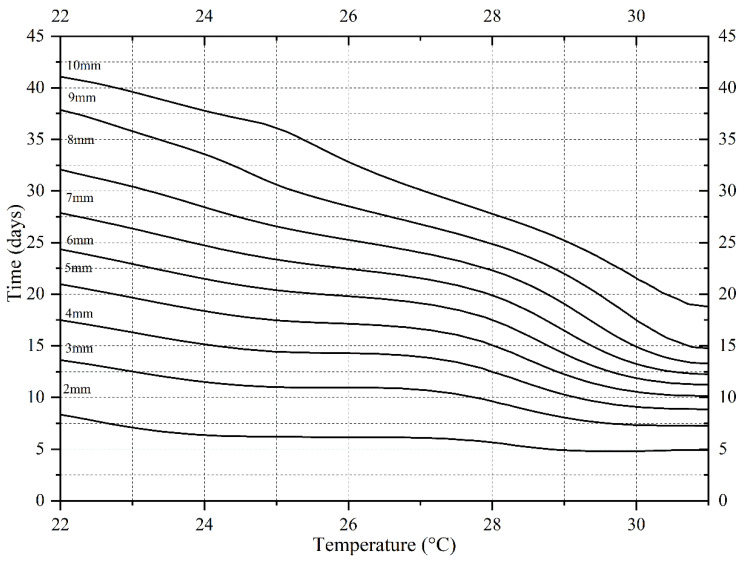
Isomegalen diagram of *Necrobia ruficollis.* Each contour represents a specific larval body length.

Based on the curve of larval body length changes and the simulation equation data in Figure 5 and Table 4, respectively, an isomegalen diagram in the temperature range between 22 and 31 °C was generated (Figure 6). Due to the extension of development time, the contour in the isomegalen diagram was abnormal at 34 °C and the data at this temperature were therefore not listed. The isomegalen diagram generated can be used for calculating the larval body length up to the peak value.

### 3.4. Larval Instar Estimation of N. ruficollis

**Figure 7:**
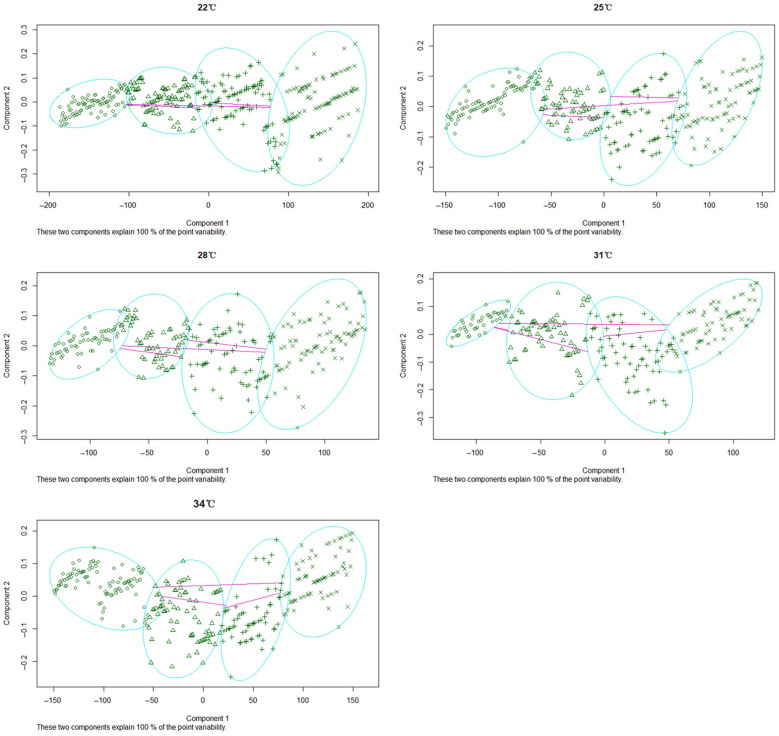
The cluster analysis diagram of head capsule width and cercus spacing at each instar of *Necrobia ruficollis* larvae under different constant temperatures. There are four instars of *Necrobia ruficollis* larvae, so the category of cluster analysis was set to four. Through cluster analysis, the larval head capsule width and cercus spacing were divided into four categories. Each blue ellipse represented one instar; ○, △, +, and × represented first, second, third, and fourth instar, respectively.

Cluster analysis of data of the head capsule width and the distance between the urogomphi of *N. ruficollis* larvae at different temperatures was conducted (Figure 7). The data were divided into four distinct elliptical regions and each one represented one instar. The result was consistent with the observation that larvae had four instars during development. The head capsule width and the distance between urogomphi of larvae in different instars could be obtained through the analysis of the data from the same category at different temperatures. The average value of larval head capsule width at the 1st instar, 2nd instar, 3rd instar, and 4th instar of *N. ruficollis* was 0.16, 0.29, 0.40, and 0.53 mm, respectively, while urogomphi distance was 0.14, 0.28, 0.44, and 0.60 mm, respectively (Table 5). Through the application of linear discriminant analysis, two indexes, including head width and pronotum of *N. ruficollis* larvae, were used to establish the discriminant function for instar: *y* = 11.24 × *f*1 × 6.32 × *f*2.

The measured values of head capsule width and pronotum width for training samples were substituted into this discriminant function and the prediction and classification were conducted on observed samples according to the discriminant function. The total discriminant accuracy of the discriminant formula was 84.4%. The discriminant accuracies of 1st-, 2nd-, 3rd-, and 4th-instar larvae were 99.44, 75.56, 59.11, and 90.96%, respectively (Table 6).

## 4. Discussion

As a storage pest, *N. ruficollis* has a wide-ranging diet, and many animal products can be used as its food [36,38]. In this study, the dried pork slices were fed to *N. ruficollis* adults, considering that the larvae would often drill into the gaps of large meat pieces, making them difficult to observe; the dried pork slices were therefore smashed into small pieces before being fed to the larvae. The pork slices were easy to obtain and process, and in this study, it was found that both the adults and larvae raised on this food exhibited good growth. Nevertheless, we were unable to evaluate whether the development data of *N. ruficollis* obtained in this study were consistent with the development pattern on a corpse under field conditions; this requires further studies which should evaluate the effects of different food types on the development of *N. ruficollis*, in order to obtain the experimental data which would better represent its development in the natural environment.

Previous studies showed that *N. ruficollis* will pupate on multiple substrates, such as existing cavities (including empty puparium of fly), sawdust, cork, and even banana peels [41], but all tended to pupate in a hidden place. In this study, the sandy soil, cotton, wood, and paper tissue were provided as the pupation substrates for larvae at the post-feeding stage, and it was found that they preferred to pupate in cotton. The larvae produced filamentous secretions that formed a slightly hardened pupal chamber and the formed pupal chamber was similar to a silkworm cocoon, but smaller and thinner. The contours of the insect body in the pupal chamber could be observed by the transmission light of the microscope. There existed a pre-pupal stage between pupal chamber formation and pupation during which the larval length gradually shortened to a point similar to the pupal length, which was followed by pupation after a period of time. If the pupal chamber was opened before the larval body length shortened, larvae would leave the pupal chamber. After crawling away, some larvae would choose to repair the damaged pupal chamber and continued to pupate, while other larvae would re-form the pupal chamber. Due to the delay in pupation time caused by the destruction of the pupal chamber, the insect body changes in the pupal chamber were observed through transmission light and the pupation time could be accurately judged by the phenomenon that the pupae would twist their abdomens. In this study, in order to record the exact adult emergence time and facilitate the observation, the pupal chambers were opened after the larvae had developed into pupae. The pupae can continue its development process and almost all pupae were emerged successfully, and we did not find the obvious effects on development velocity and eclosion rate of pupae. However, as there were no pervious data to prove the effect of opening the pupal chamber on pupal development velocity and eclosion rate, and the comparative studies were not conducted in this study, whether this operation will affect the development velocity and eclosion rate of pupae needs to be studied further. The head and thorax of a newly formed pupa is milk white and translucent, and the abdomen is covered with irregular black dark spots. With development, the insect will become colored in the order from eyes, mouthparts, and legs/wings. In the study by Novák et al. [47], the age of the pupal stage of Silphidae could be estimated through quantifying the changes in compound eye color of the pupa. In future research, observations of external morphological changes in *N. ruficollis* pupae could provide a basis for pupal age estimation.

The development rate of *N. ruficollis* accelerated with an increasing temperature between 22 and 31 °C, while the overall development rate decreased at 34 °C. At 31 °C, the minimum development duration of *N. ruficollis* from egg to adult was 42.33 days, while it was 52.33 days at 34 °C, which was an increase compared to that observed at 31 °C. This indicates that 34 °C might be the maximum tolerance temperature of *N. ruficollis*. This was consistent with the upper lethal developmental threshold temperature (34.94 °C) inferred by the Curvilinear crystal model (Optim SSI). Compared with D_0_ (14.51 °C) obtained by the revised linear regression model proposed by Ikemoto and Takai [45], a similar *T_L_* (14.61 °C) was estimated by a nonlinear model of Shi et al. [46] (the variation was less than 0.7%). The development rate of theses insects generated a bell-shaped curve. The linear thermal summation model is only applicable to the linear range of growth rate change and is not applicable when the temperature change is close to the lower and upper developmental threshold. This study adopted the linear model proposed by Ikemoto and Takai [45], who put forward their opinions on model modification. Data points at critical temperatures could not be included in the linear array of points, which results in an uncertainty in the optimum temperature range as well as an unreliable estimation of parameters [45]. Therefore, nonlinear data that are outside the scope of the linear model should be removed. Thus, in this study, the nonlinear data at 34°C were removed during the linear regression analysis according to the recommendation of Ikemoto and Takai [45]. The curvilinear modeling was able to simulate the growth rate of *N. ruficollis* at 22–34 °C, including the nonlinear relationship at 34 °C; it can therefore be used to calculate lower and upper thermal thresholds as well as the optimum temperature for development. Nonlinear models have been studied extensively in recent years. The SSI model selected in this study can calculate the optimal temperature when the enzyme activity is the highest (the probability of an enzyme being in its active state is maximal), but not the optimum temperature when the growth rate is the fastest.

Although many studies have emphasized the value of *N. ruficollis* in forensic investigations, no development data about this species were available. Compared to other sarcosaprophagous beetles, the development time of *N. ruficollis* is much longer. For example, at 25 °C, the development times for *Euspilotus azureus* (Sahlberg, 1823) (Coleoptera: Histeridae) [30], *Thanatophilus sinuatus* (Fabricius, 1775) (Coleoptera: Silphidae) [48], *Dermestes frischii* (Kugelan, 1792) (Coleoptera: Dermestidae) [27], and *Omosita colon* (Linnaeus, 1758) (Coleoptera: Nitidulidae) [26] from eggs to eclosion are 31.5, 24.8, 36.0, and 34.9 days, respectively, while *N. ruficollis* requires 62.7 days to complete one generation development at this temperature. Compared to the closely related species of *N. rufipes*, the development duration of *N. rufipes* from egg to adult at 25 °C is 66.2 days [43], which is similar to the development time of *N. ruficollis*. *Necrobia ruficollis* and *N. rufipes* are related species, and are very similar in morphological, genetic, and ecological attributes, which may explain the similarity in their development data.

*Necrobia ruficollis* is one of the last insect species to arrive at the corpse. When the immature *N. ruficollis* is prevalent on the corpse, most of the flies that colonize the cadaver early, such as blow flies, have accomplished one generation of development and have left the corpse. As a result, research on *N. ruficollis* can improve the accuracy of PMI estimation in the “post-fly stage” and provide a scientific basis for PMI estimations for highly decomposed and skeletonized corpses. In addition, the immature stage of *N. ruficollis* is longer than that of flies. The longer development time of *N. ruficollis* can further expand the estimation range of the PMI and corroborate the indexes of flies and other beetles, possibly obtaining more accurate PMI results. Simultaneously, in many succession experiments around the world, it was found that the time for *N. ruficollis* to colonize a corpse was different with the seasons [15,16,49], which means that it is of great importance to determine the colonization time in different seasons in corresponding areas.

Sarcosaprophagous beetles have a longer life history and their reproduction rates are far lower than that of flies; it is thus difficult to collect enough eggs for continuous and intensive sampling in a short time. Considering that the study required a high sample size, in this study, in vivo photography was applied at specific times to obtain developmental and morphological data. To reduce disruption to larvae, we were very careful during in vivo photography and the shooting time was also shortened as much as possible, but there still exists the risk that the data obtained in this study differs from the growth and development that would be observed in the natural environment. In particular, the research of Frątczak-Łagiewska and Matuszewski [34] pointed out that the larval size from in vivo measurements would be smaller, and the data obtained through in vivo measurements might overestimate the actual age of larvae. In future research, a sampling method with the minimum impact on larval development should be investigated, and the effects of in vivo measurement on larval development should be further studied.

In most forensic entomology cases, the temperature experienced by insects fluctuates rather than remaining constant [6]. Hence, theoretically, the age of immature stage should be estimated using development data obtained under fluctuating temperatures. However, fluctuation of temperatures is hard to model, as they can manifest in a variety of ways even with the same average temperature. In practice, forensic entomologists usually average the fluctuating environmental temperature to which the corpses are subjected, and then used the data of same constant temperature to estimate the PMI. Several studies have been conducted on the effects of constant and fluctuating temperatures on insect development, and different researchers hold different opinions. Milosavljevic et al. [50] and McCalla et al. [51] found that compared with a constant temperature, a fluctuating temperature had a significant impact on the development rates of Encyrtidae and Eulophidae. Parasitoids reared under fluctuating profiles at low average temperatures developed faster and survived for longer when compared to those reared under constant temperatures. In contrast, high average fluctuating temperatures produced parasitoids with an extended developmental period and reduced longevity. Wu et al. studied the development duration of *Trichogramma dendrolimi* (Matsumura, 1926) (Hymenoptera: Trichogrammatidae) and *Diaphania indica* (Saunders, 1851) (Lepidoptera: Crambidae) under various constant and fluctuating temperature regimes, and results revealed that a fluctuating temperature did not influence the instantaneous rate of development (the variation is less than 4%), and the thermal inputs for completing development did not lead to significant differences, or were similar between constant and fluctuating temperature regimes [52]. In the study of the influence of fluctuating temperature on Coleoptera, Hagstrum and Leach [53] found that development time of *Tribolium castancum* (Herbst,1797) (Coleoptera: Tenebrionidae) and *Trogoderma iuclusum* (LeConte, 1854) (Coleoptera: Dermestidae) was reduced by about 9–12% when beetles were exposed to sinusoidal fluctuating temperatures rather than a constant temperature. However, *Sitophilus oryzac* (L. 1763) (Coleoptera: Curculionidae) showed no significant difference in the effect of the two temperature conditions on development time. Another study by Hagstrum and Milliken [54] showed that developmental time data collected at constant temperatures poorly predicted developmental times of red flour beetle, *T. castaneum*, and 16 other species at fluctuating temperatures over a broad range of mean temperatures or amplitudes of fluctuating temperatures. Developmental times at constant temperatures tended to be shorter above 25–30 °C temperature range and longer below this range than at fluctuating temperatures with the same means. In forensic entomology, Byrd and Butler have carried out studies on the development of flies of forensic significance under fluctuating temperature conditions, including *Chrysomya rufifacies* (Macquart, 1844) (Diptera: Calliphoridae) [55], *Cochliomyia macellaria* (Fabricius, 1775) (Diptera: Calliphoridae) [56], and *Sarcophaga haemorrhoidalis* (Fallen, 1871) (Diptera: Sarcophagidae) [57]. In this paper, we provide the development data of *N. ruficollis* under constant temperature, which may differ from the development pattern of this species under natural conditions, highlighting a limitation of this study in forensic medicine practice. Future studies should compare the development rates of the species at different fluctuations and constant temperatures to enable more accurate PMI.

In addition, our choice of experimental temperature range has limitations. According to our previous studies on insect succession in the field and in other areas, we found that the *N. ruficollis* tends to appear in warm seasons [15,16]. In addition, Hu et al. [43] found that the death rate of its closely related species *N. rufipes* at the constant temperatures of 19 °C was 100%. Considering that *N. rufipes* and *N. ruficollis* are usually present in cadavers at the same time, the same thermal biology characteristic may apply. Therefore, the experimental temperature of 19 °C or lower was not set in this study. However, we should take into account that the *N. ruficollis* and *N. rufipes* are not the same species, and there may be significant differences in the developmental threshold temperatures, which will be verified by further experiments in the future.

## 5. Conclusions

*Necrobia ruficollis* is a forensically important species which has great significance and potential for estimating the PMI_min_. This study provides developmental data of *N. ruficollis* subjected to five constant temperatures. The longest developmental time was observed at 16 °C (92.00 ± 2.94–96.33 ± 0.94 days) and the shortest at 31 °C (42.33 ± 2.05–57.00 ± 1.63 days). At 34 °C, *N. ruficollis* showed developmental delays. We generated temperature-dependent developmental models for *N. ruficollis*, including isomorphen diagram, isomegalen diagram, and linear and nonlinear thermal summation models. The development threshold temperature D_0_ and thermal summation constant K, which are frequently used indicators for estimating PMI_min_, were obtained by linear regression analysis as 14.51 ± 0.52 °C and 684.12 ± 33.85 degree days. The lower developmental threshold, intrinsic optimum temperature, and upper lethal developmental threshold temperature estimated by the nonlinear model were 14.61, 25.90, and 34.94 °C, respectively. In addition, the head capsule width and pronotum width at different instars were determined by cluster analysis. Classifiers were created and validated by linear discriminant analysis. This study provides new development data of *N. ruficollis* for forensic entomology.

## Figures and Tables

**Table 1:** Mean duration (days ± SD) of different developmental stages of *Necrobia ruficollis* under laboratory conditions with five temperatures, a photoperiod of 12:12 h L:D, and humidity of 70%.

Developmental Stages		Temperature (°C)				
		22	25	28	31	34
Hatching	min	6.14 ± 0.16	4.97 ± 0.02	3.99 ± 0.09	2.97 ± 0.04	2.91 ± 0.11
	50% individuals	6.28 ± 0.20	5.11 ± 0.04	4.15 ± 0.12	3.05 ± 0.04	2.94 ± 0.14
	max	6.72 ± 0.39	5.27 ± 0.08	4.25 ± 0.07	3.11 ± 0.08	3.11 ± 0.08
1st ecdysis	min	16.33 ± 0.47	13.00 ± 0.00	10.00 ± 0.00	8.33 ± 0.47	7.67 ± 0.47
	50% individuals	18.67 ± 0.94	14.00 ± 0.81	11.33 ± 0.47	9.00 ± 0.82	9.33 ± 0.47
	max	21.33 ± 2.05	15.00 ± 0.82	12.24 ± 0.55	10.33 ± 1.25	10.33 ± 0.47
2nd ecdysis	min	25.33 ± 0.94	21.67 ± 0.94	16.67 ± 0.47	13.67 ± 1.25	14.33 ± 0.94
	50% individuals	28.33 ± 1.25	23.33 ± 1.24	18.00 ± 0.00	14.67 ± 1.25	16.33 ± 2.05
	max	30.33 ± 1.89	24.67 ± 1.25	29.00 ± 0.00	15.67 ± 1.25	18.00 ± 2.16
3rd ecdysis	min	36.33 ± 0.94	29.00 ± 0.82	23.67 ± 0.47	19.00 ± 1.41	20.33 ± 2.05
	50% individuals	42.00 ± 0.82	31.33 ± 0.93	26.00 ± 1.63	21.00 ± 1.41	23.33 ± 4.78
	max	50.33 ± 3.09	33.00 ± 1.41	27.33 ± 1.69	22.67 ± 1.89	26.67 ± 5.18
Chamber formation	min	63.00 ± 2.94	41.33 ± 2.05	38.33 ± 3.09	30.67 ± 0.94	43.33 ± 6.85
	50% individuals	70.00 ± 0.00	46.00 ± 3.27	51.00 ± 5.72	35.00 ± 2.16	44.67 ± 8.73
	max	82.00 ± 3.56	57.33 ± 9.10	68.67 ± 4.99	42.33 ± 4.11	47.67 ± 6.60
Pupation	min	79.00 ± 3.74	52.33 ± 2.05	46.00 ± 0.82	36.00 ± 1.63	47.00 ± 7.12
	50% individuals	80.33 ± 2.49	59.67 ± 1.70	57.33 ± 3.40	40.67 ± 0.47	49.67 ± 8.05
	max	85.67 ± 3.86	66.33 ± 6.85	74.67 ± 3.40	49.67 ± 1.70	52.33 ± 6.18
Adult emergence	Min	92.00 ± 2.94	62.67 ± 2.05	51.33 ± 2.05	42.33 ± 2.05	52.33 ± 7.93
	50% individuals	93.00 ± 1.63	70.67 ± 0.94	65.33 ± 3.40	47.33 ± 0.94	56.67 ± 8.73
	max	96.33 ± 0.94	76.33 ± 6.13	82.67 ± 2.87	57.00 ± 1.63	59.00 ± 7.12

**Table 2:** Developmental threshold temperatures (D_0_) and thermal summation constants (K) for different development stages. The coefficient of determination (R^2^) of thermal summation models was calculated according to the method described by Ikemoto and Takai [45].

Developmental Stage	K ± SE (Degree Days)	D0 ± SE (°C)	R2
Hatching	59.07 ± 3.17	12.71 ± 0.72	0.96
1st ecdysis	159.34 ± 7.61	12.41 ± 0.66	0.96
2nd ecdysis	265.37 ± 22.57	12.08 ± 1.16	0.90
3rd ecdysis	361.42 ± 23.46	12.31 ± 0.84	0.95
Chamber formation	505.58 ± 48.64	13.96 ± 1.08	0.94
Pupation	588.72 ± 37.61	14.51 ± 0.67	0.97
Adult emergence	684.12 ± 33.85	14.51 ± 0.52	0.99

SE: Standard error.

**Table 3:** Parameter estimates for different developmental stages of *Necrobia ruficollis* according to the curvilinear thermodynamic model (Optim SSI) of Shi et al. [46].

Parameter (Unit)	Hatching	1st Ecdysis	2nd Ecdysis	3rd Ecdysis	Chamber Formation	Pupation	Adult Emergence
*T_Φ_* (°C)	23.32	23.41	23.13	22.91	25.80	25.98	25.90
*ρ_Φ_* (day^−1^)	0.18	0.69	0.41	0.29	0.30	0.19	0.17
Δ*H_A_* (cal/mol)	1.36 × 10^4^	1.33 × 10^4^	1.38 × 10^4^	1.30 × 10^4^	1.42 × 10^4^	1.53 × 10^4^	1.54 × 10^4^
Δ*H_L_* (cal/mol)	−1.02 × 10^6^	−1.49 × 10^5^	−5.78 × 10^5^	−2.03 × 10^5^	−8.11 × 10^4^	−7.82 × 10^4^	−7.47 × 10^4^
Δ*H_H_* (cal/mol)	5.41 × 10^5^	1.21 × 10^5^	5.22 × 10^5^	1.84 × 10^5^	1.24 × 10^5^	1.17 × 10^5^	1.07 × 10^5^
*T_L_* (°C)	13.54	13.22	13.69	12.60	14.38	14.67	14.61
*T_H_* (°C)	34.32	36.63	34.27	35.08	34.44	34.72	34.94
χ^2^	4.32 × 10^−^^3^	2.98 × 10^−^^3^	2.05 × 10^−^^3^	2.52 × 10^−^^3^	2.12 × 10^−^^3^	1.01 × 10^−^^3^	6.91 × 10^−^^4^
R^2^	0.984	0.965	0.958	0.914	0.890	0.947	0.956

**Table 4:** Equations, degrees of freedom (df), and coefficient of determination (R^2^) of the relationship between time after hatching (*T*) (day) and the body length of *Necrobia ruficollis* larvae (*L*) (mm) at five constant temperatures.

Temperature (°C)	Simulation Equation	df	R^2^
22	*L* = −2.695 × 10^−4^*T*^3^ + 0.018*T*^2^ − 0.104*T* + 1.751	761	0.869
25	*L* = −3.589 × 10^−4^*T*^3^ + 0.021*T*^2^ − 0.066*T* + 1.668	641	0.882
28	*L* = −4.418 × 10^−4^*T*^3^ + 0.025*T*^2^ − 0.048*T* + 1.522	491	0.879
31	*L* = −1.040 × 10^−3^*T*^3^ + 0.050*T*^2^ − 0.176*T* + 1.605	446	0.857
34	*L* = −3.778 × 10^−4^*T*^3^ + 0.018*T*^2^ + 0.066*T* + 1.287	551	0.785

**Table 5:** Means (±SD) and range of the widths of head capsule (mm) and the distance of urogomphi (mm) at each instar, obtained by cluster analysis.

Morphological Indexes	Instar	Mean ± SD	Range	Sample Size
The widths of head capsule	1st	0.16 ± 0.04	0.05–0.36	360
	2nd	0.29 ± 0.06	0.17–0.44	270
	3rd	0.40 ± 0.09	0.23–0.62	269
	4th	0.53 ± 0.09	0.32–0.83	586
The distance of urogomphi	1st	0.14 ± 0.05	0.07–0.25	360
	2nd	0.28 ± 0.10	0.14–0.60	270
	3rd	0.44 ± 0.13	0.20–0.79	269
	4th	0.60 ± 0.15	0.27–0.91	586

**Table 6:** Classification matrix for test larvae of *Necrobia ruficollis*.

Instar	Sample Size	Prediction of Classification				Precision Rate
		1st	2nd	3rd	4th	
1st	360	358	2	0	0	99.44%
2nd	270	24	204	42	0	75.56%
3rd	269	0	49	159	61	59.11%
4th	586	0	0	53	533	90.96%
Total	1485	382	255	254	594	84.44%

## Data Availability

Not applicable.

## References

[1] Huntington T.E., Weidner L.M., Hall R.D., Byrd J.H., Tomberlin J.K. (2019). Introduction: Current Perceptions and Status of Forensic Entomology. Forensic Entomology: The Utility of Arthropods in Legal Investigations.

[2] Wang Y., Wang Y.H., Wang M., Xu W., Zhang Y.N., Wang J.F. (2021). Forensic entomology in China and its challenges. Insects.

[3] Dekeirsschieter J., Frederickx C., Verheggen F.J., Boxho P., Haubruge E. (2013). Forensic entomology investigations from doctor Marcel Leclercq (1924–2008): A review of cases from 1969 to 2005. J. Med. Entomol..

[4] Kulshrestha P., Satpathy D.K. (2001). Use of beetles in forensic entomology. Forensic Sci. Int..

[5] Castro C.P.E., García M.D., Silva P.D.M., Silva I.F.E., Serrano A. (2013). Coleoptera of forensic interest: A study of seasonal community composition and succession in Lisbon, Portugal. Forensic Sci. Int..

[6] Amendt J., Richards C.S., Campobasso C.P., Zehner R., Hall M.J.R. (2011). Forensic entomology: Applications and limitations. Forensic Sci. Med. Pathol..

[7] Greenberg B., Kunich J.C. (2002). Entomology and the Law: Flies as Forensic Indicators.

[8] Gruner S.V., Slone D.H., Capinera J.L., Turco M.P. (2017). Development of the oriental latrine fly, *Chrysomya megacephala* (Diptera: Calliphoridae), at five constant temperatures. J. Med. Entomol..

[9] Kotzé Z., Villet M.H., Weldon C.W. (2015). Effect of temperature on development of the blowfly, *Lucilia cuprina* (Wiedemann) (Diptera: Calliphoridae). Int. J. Legal Med..

[10] Russo A., Cocuzza G.E., Vasta M.C., Simola M., Virone G. (2006). Life fertility tables of *Piophila casei* L. (Diptera: Piophilidae) reared at five different temperatures. Environ. Entomol..

[11] Grassberger M., Reiter C. (2002). Effect of temperature on development of the forensically important holarctic blow fly *Protophormia terraenovae* (Robineau-Desvoidy) (Diptera: Calliphoridae). Forensic Sci. Int..

[12] Kocarek P. (2003). Decomposition and Coleoptera succession on exposed carrion of small mammal in Opava, the Czech Republic. Eur. J. Soil Biol..

[13] Souza A.M.D., Linhares A.X. (1997). Diptera and Coleoptera of potential forensic importance in southeastern Brazil: Relative abundance and seasonality. Med. Vet. Entomol..

[14] Mayer A.C.G., Vasconcelos S.D. (2013). Necrophagous beetles associated with carcasses in a semi-arid environment in Northeastern Brazil: Implications for forensic entomology. Forensic Sci. Int..

[15] Abouzied E.M. (2014). Insect colonization and succession on rabbit carcasses in southwestern mountains of the Kingdom of Saudi Arabia. J. Med. Entomol..

[16] Voss S.C., Cook D.F., Dadour I.R. (2011). Decomposition and insect succession of clothed and unclothed carcasses in Western Australia. Forensic Sci. Int..

[17] Özdemir S., Sert O. (2009). Determination of Coleoptera fauna on carcasses in Ankara province, Turkey. Forensic Sci. Int..

[18] Midgley J.M., Richards C.S., Villet M.H., Amendt J., Goff M.L., Campobasso C.P., Grassberger M. (2009). The Utility of Coleoptera in Forensic Investigations. Current Concepts in Forensic Entomology.

[19] Amendt J., Campobasso C.P., Gaudry E., Reiter C., LeBlanc H.N., Hall M.J.R. (2007). Best practice in forensic entomology—standards and guidelines. Int. J. Legal Med..

[20] Bonacci T., Mendicino F., Bonelli D., Carlomagno F., Curia G., Scapoli C., Pezzi M. (2021). Investigations on arthropods associated with decay stages of buried animals in Italy. Insects.

[21] Grassberger M., Frank C. (2004). Initial study of arthropod succession on pig carrion in a central European urban habitat. J. Med. Entomol..

[22] Velásquez Y., Viloria A.L. (2009). Effects of temperature on the development of the Neotropical carrion beetle *Oxelytrum discicolle* (Brullé, 1840) (Coleoptera: Silphidae). Forensic Sci. Int..

[23] Frątczak K., Matuszewski S. (2014). Instar determination in forensically useful beetles *Necrodes littoralis* (Silphidae) and *Creophilus maxillosus* (Staphylinidae). Forensic Sci. Int..

[24] Lin S.W., Shiao S.F. (2013). Life history data on the fly parasitoids *Aleochara nigra* Kraatz and *A. asiatica* Kraatz (Coleoptera: Staphylinidae), and their potential application in forensic entomology. Forensic Sci. Int..

[25] Frątczak-Łagiewska K., Matuszewski S. (2018). Sex-specific developmental models for *Creophilus maxillosus* (L.) (Coleoptera: Staphylinidae): Searching for larger accuracy of insect age estimates. Int. J. Legal Med..

[26] Wang Y., Wang M., Hu G.L., Xu W., Wang Y.H., Wang J.F. (2020). Temperature-dependent development of *Omosita colon* at constant temperature and its implication for PMI_min_ estimation. J. Forensic Legal Med..

[27] Lambiase S., Murgia G., Sacchi R., Ghitti M., di Lucia V. (2018). Effects of different temperatures on the development of *Dermestes frischii* and *Dermestes undulatus* (Coleoptera, Dermestidae): Comparison between species. J. Forensic Sci..

[28] Zanetti N.I., Visciarelli E.C., Centeno N.D. (2016). The effect of temperature and laboratory rearing conditions on the development of *Dermestes maculatus* (Coleoptera: Dermestidae). J. Forensic Sci..

[29] Jakubec P. (2016). Thermal summation model and instar determination of all developmental stages of necrophagous beetle, *Sciodrepoides watsoni* (Spence) (Coleoptera: Leiodidae: Cholevinae). PeerJ.

[30] Caneparo M.F.C., Fischer M.L., Almeida L.M. (2017). Effect of temperature on the life cycle of *Euspilotus azureus* (Coleoptera: Histeridae), a predator of forensic importance. Florida Entomol..

[31] Zanetti N.I., Costantino A., Lazzarini N., Ferrero A.A., Centeno N.D. (2021). *Dermestes maculatus* (Coleoptera: Dermestidae) development under fluoxetine effect using two drug administration models. J. Forensic Sci..

[32] Qubaiová J., Jakubec P., Montoya-Molina S., Novák M., Šuláková H. (2021). Influence of diet on development and survival of *Thanatophilus rugosus* (Coleoptera: Silphidae). J. Med. Entomol..

[33] Fontenot E.A., Arthur F.H., Hartzer K.L. (2015). Effect of diet and refugia on development of *Dermestes maculatus* DeGeer reared in a laboratory. J. Pest Sci..

[34] Frątczak-Łagiewska K., Matuszewski S. (2019). The quality of developmental reference data in forensic entomology: Detrimental effects of multiple, in vivo measurements in *Creophilus maxillosus* L. (Coleoptera: Staphylinidae). Forensic Sci. Int..

[35] Zanetti N.I., Ferrero A.A., Centeno N.D. (2020). Type of wood and larval density: Two factors to consider in *Dermestes maculatus* (Coleoptera: Dermestidae) pupation. Rev. Soc. Entomol. Arge..

[36] Zhao Z.G. (1994). Household insects and their regulation. J. Bijie Teach. Coll..

[37] Lin W.J., Lin J.F., Lin X., Ruan Q. (1994). Control of mites in fish meal by aluminum phosphide interval fumigation. Grain. Storage.

[38] Meng F.M., Ren L.P., Shang Y.J., Chen W., Cai J.F., Guo Y.D. (2019). The complete mitochondria genome of a forensic related beetle, *Necrobia ruficollis* (Fabricius, 1775). Mitochondrial DNA B.

[39] Early M., Goff M.L. (1986). Arthropod succession patterns in exposed carrion on the island of Oahu, Hawaiian-Islands, USA. J. Med. Entomol..

[40] Lyu Z., Wan L.H., Yang Y.Q., Tang R., Xu L.Z. (2016). A checklist of beetles (Insecta, Coleoptera) on pig carcasses in the suburban area of southwestern China: A preliminary study and its forensic relevance. J. Forensic Legal Med..

[41] Scott H. (1919). Notes on the biology of *Necrobia ruficollis* fabr. (Coleoptera: Cleridae). Ann. Appl. Biol..

[42] Yadav J.S., Dange M.P. (1989). On the cytology of two species of *Necrobia* (Oliv.) (Coleoptera: Cleridae). Genome.

[43] Hu G.L., Wang M., Wang Y., Tang H.H., Chen R.F., Zhang Y.N., Zhao Y.L., Jin J.Y., Wang Y.F., Wu M.W. (2020). Development of *Necrobia rufipes* (De Geer, 1775) (Coleoptera: Cleridae) under constant temperatures and its implication in forensic entomology. Forensic Sci. Int..

[44] Zhang S.F., Shi S.F., Shi Z.W., Xue G.H. (2008). Atlas of Beetles Associated with Stored Products.

[45] Ikemoto T., Takai K. (2000). A new linearized formula for the law of total effective temperature and the evaluation of line-fitting methods with both variables subject to error. Environ. Entomol..

[46] Shi P., Ikemoto T., Egami C., Sun Y., Ge F. (2011). A modified program for estimating the parameters of the SSI model. Environ. Entomol..

[47] Novák M., Frątczak-Łagiewska K., Mądra-Bielewicz A., Matuszewski S. (2020). Eye-background contrast as a quantitative marker for pupal age in a forensically important carrion beetle *Necrodes littoralis* L. (Silphidae). Sci. Rep.-UK.

[48] Montoya-Molina S., Jakubec P., Qubaiová J., Novák M., Šuláková H., Růžička J. (2021). Developmental models of the forensically important carrion beetle, *Thanatophilus sinuatus* (Coleoptera: Silphidae). J. Med. Entomol..

[49] Wang J.F., Li Z.G., Chen Y.C., Chen Q.S., Yin X.H. (2008). The succession and development of insects on pig carcasses and their significances in estimating PMI in south China. Forensic Sci. Int..

[50] Milosavljevic I., McCalla K.A., Ratkowsky D.A., Hoddle M.S. (2019). Effects of constant and fluctuating temperatures on development rates and longevity of *Diaphorencyrtus aligarhensis* (Hymenoptera: Encyrtidae). J. Econ. Entomol..

[51] McCalla K.A., Keçeci M., Milosavljević I., Ratkowsky D.A., Hoddle M.S. (2019). The influence of temperature variation on life history parameters and thermal performance curves of *Tamarixia radiata* (Hymenoptera: Eulophidae), a parasitoid of the Asian citrus psyllid (Hemiptera: Liviidae). J. Econ. Entomol..

[52] Wu X.J., Liu S.S., Zheng Z.L. (1994). The influence of variable temperature upon rate of development in two insects. Entomol. Knowl..

[53] Hagstrum D.W., Leach C.E. (1973). Role of constant and fluctuating temperatures in determining development time and fecundity of 3 species of stored-products Coleoptera. Ann. Entomol. Soc. Am..

[54] Hagstrum D.W., Milliken G.A. (1991). Modeling differences in insect developmental times between constant and fluctuating temperatures. Ann. Entomol. Soc. Am..

[55] Byrd J.H., Butler J.F. (1997). Effects of temperature on *Chrysomya rufifacies* (Diptera: Calliphoridae) development. J. Med. Entomol..

[56] Byrd J.H., Butler J.F. (1996). Effects of temperature on *Cochliomyia macellaria* (Diptera: Calliphoridae) development. J. Med. Entomol..

[57] Byrd J.H., Butler J.F. (1998). Effects of temperature on *Sarcophaga haemorrhoidalis* (Diptera: Sarcophagidae) development. J. Med. Entomol..

